# Developing and Implementing a Web-Based Branching Logic Survey to Support Psychiatric Crisis Evaluations of Individuals With Developmental Disabilities: Qualitative Study and Evaluation of Validity

**DOI:** 10.2196/50907

**Published:** 2024-03-29

**Authors:** Deborah A Bilder, Mariah Mthembu, Whitney Worsham, Patricia Aguayo, Jacob R Knight, Steven W Deng, Tejinder P Singh, John Davis

**Affiliations:** 1 University of Utah Huntsman Mental Health Institute Salt Lake City, UT United States; 2 University of Utah School of Medicine Salt Lake City, UT United States; 3 University of Utah Salt Lake City, UT United States

**Keywords:** developmental disabilities, disruptive behavior, psychiatric comorbidity, web-based, psychiatric crisis, disability, mental health, behavioral crises, intervention, general population, screening, accuracy, mood disorder, sources of distress, autism, intellectual disability

## Abstract

**Background:**

Individuals with developmental disabilities (DD) experience increased rates of emotional and behavioral crises that necessitate assessment and intervention. Psychiatric disorders can contribute to crises; however, screening measures developed for the general population are inadequate for those with DD. Medical conditions can exacerbate crises and merit evaluation. Screening tools using checklist formats, even when designed for DD, are too limited in depth and scope for crisis assessments. The Sources of Distress survey implements a web-based branching logic format to screen for common psychiatric and medical conditions experienced by individuals with DD by querying caregiver knowledge and observations.

**Objective:**

This paper aims to (1) describe the initial survey development, (2) report on focus group and expert review processes and findings, and (3) present results from the survey’s clinical implementation and evaluation of validity.

**Methods:**

Sources of Distress was reviewed by focus groups and clinical experts; this feedback informed survey revisions. The survey was subsequently implemented in clinical settings to augment providers’ psychiatric and medical history taking. Informal and formal consults followed the completion of Sources of Distress for a subset of individuals. A records review was performed to identify working diagnoses established during these consults.

**Results:**

Focus group members (n=17) expressed positive feedback overall about the survey’s content and provided specific recommendations to add categories and items. The survey was completed for 231 individuals with DD in the clinical setting (n=161, 69.7% men and boys; mean age 17.7, SD 10.3; range 2-65 years). Consults were performed for 149 individuals (n=102, 68.5% men and boys; mean age 18.9, SD 10.9 years), generating working diagnoses to compare survey screening results. Sources of Distress accuracy rates were 91% (95% CI 85%-95%) for posttraumatic stress disorder, 87% (95% CI 81%-92%) for anxiety, 87% (95% CI 81%-92%) for episodic expansive mood and bipolar disorder, 82% (95% CI 75%-87%) for psychotic disorder, 79% (95% CI 71%-85%) for unipolar depression, and 76% (95% CI 69%-82%) for attention-deficit/hyperactivity disorder. While no specific survey items or screening algorithm existed for unspecified mood disorder and disruptive mood dysregulation disorder, these conditions were caregiver-reported and working diagnoses for 11.7% (27/231) and 16.8% (25/149) of individuals, respectively.

**Conclusions:**

Caregivers described Sources of Distress as an acceptable tool for sharing their knowledge and insights about individuals with DD who present in crisis. As a screening tool, this survey demonstrates good accuracy. However, better differentiation among mood disorders is needed, including the addition of items and screening algorithm for unspecified mood disorder and disruptive mood dysregulation disorder. Additional validation efforts are necessary to include a more geographically diverse population and reevaluate mood disorder differentiation. Future study is merited to investigate the survey’s impact on the psychiatric and medical management of distress in individuals with DD.

## Introduction

### Background

Individuals with developmental disabilities (DD) such as autism and intellectual disability (ID) experience mental health crises more frequently than the general population [1,2]. A broad range of psychiatric and medical conditions can contribute to the agitation, aggression, and self-injury that often characterize these crises [3-10]. Rates of anxiety (20%-77%), depression (10%-20%), expansive mood and bipolar disorder (5%-11%), and psychosis (5%-10%) among individuals with autism exceed those in neurotypical individuals [11-19]. Elevated rates of psychiatric disorders have also been identified in individuals with ID, notably for unspecified psychosis (4.8%), schizophrenia (3.9%), and bipolar disorder (8%) [20-22]. A history of trauma or abuse should also be considered in individuals with DD presenting in crisis [23].

When psychiatric and medical conditions are recognized as factors contributing to a person’s mental health crisis, clear long-term treatment targets emerge. Nevertheless, for those with DD, co-occurring medical and psychiatric conditions are often unrecognized, leaving them vulnerable to experiencing diagnostic overshadowing. Diagnostic overshadowing occurs when disruptive behaviors in individuals with DD are attributed to their disability without consideration of other potential medical or psychiatric conditions that could contribute to their behavioral presentation [24].

Self-, parent-, and caregiver-report mental health questionnaires provide an efficient means of screening for common psychiatric conditions in the neurotypical population. However, for those with DD, self-report questionnaires may be impeded by communication deficits or a limited capacity to reflect on internal experiences. Parent- and caregiver-report questionnaires normed in typically developing children may also provide inadequate mental health screening for those with ID because they often include items that are inapplicable to children with minimal language ability, exclude severe conditions that disproportionately affect children with DD (eg, mania and psychosis), and overlook the individualized manner in which psychiatric symptoms manifest in this population [20,25-27].

The American Psychiatric Association and the National Association for the Dually Diagnosed published the *Diagnostic Manual–Intellectual Disability* in 2007, and subsequently, in 2016, the second edition (*Diagnostic Manual–Intellectual Disability–Second Edition*; *DM-ID-2*) [28,29]. These texts adapt the *Diagnostic and Statistical Manual of Mental Disorders* criteria to reflect their presentation in individuals with ID. The Psychopathology Instrument for Mentally Retarded Adults and the Psychiatric Assessment Schedule for Adults with Developmental Disabilities (PAS-ADD) operationalize adapted diagnostic criteria into structured interviews to provide a framework through which to identify psychiatric conditions in this population [30,31]. These interviews are quite lengthy and require training to administer. Even as an abbreviated semistructured interview, the Mini PAS-ADD Clinical Interview takes approximately 45 minutes to complete [32]. Existing parent- and caregiver-report psychiatric screening tools for individuals with ID create a more efficient and practical means of collecting information [33-36]; yet, the checklist format of parent- and caregiver-report questionnaires limits depth and scope, both of which are necessary when evaluating crises in a population with complex medical and mental health needs. In addition, there is a great need for the inclusion of items that query symptoms of common medical conditions (eg, epilepsy, gastrointestinal disorders, and poor dentition) that manifest with agitation and aggression and occur more frequently in individuals with DD [3,37,38].

Sources of Distress is a survey developed for parents and caregivers (hereinafter collectively referred to as *caregivers*) that uses a web-based branching logic format to screen for mental health and medical conditions among individuals with DD who present in crisis. This tool informs the care of individuals experiencing distress and is intended for use when the severity or persistence of disruptive behavior prompts the consideration of medication intervention. Screening information endorsed by caregivers is organized into relevant psychiatric and medical categories within a report. This report (Multimedia Appendix 1 [39]) is developed for the caregiver and can subsequently facilitate their shared decision-making process with health care providers as specific underlying conditions are evaluated. Sources of Distress aims to minimize diagnostic overshadowing and optimize the ability of the caregiver and the provider to recognize the presence of psychiatric and medical conditions that merit targeted intervention. The web-based branching logic format is adaptive in nature—optimizing caregiver and health care provider convenience and efficiency and minimizing caregiver burden for survey completion [40].

### Objectives

This paper aims to (1) describe the initial development of Sources of Distress; (2) report on the findings from focus group evaluations and expert reviews and indicate how this feedback shaped the subsequent version of the survey; and (3) present the results from the evaluation of validity for Sources of Distress after its implementation in the clinical setting. The *Methods* and *Results* sections are divided into 3 subsections (apart from the *Ethical Considerations* section in *Methods*) corresponding to the development, initial evaluation, and clinical implementation phases of Sources of Distress.

## Methods

### Ethical Considerations

The University of Utah Institutional Review Board approved focus group activities for Sources of Distress content validation (IRB_00111975). Focus group participants provided informed consent and received compensation for their time in the form of an Amazon gift card worth US $50. The University of Utah Institutional Review Board approved with a waiver of consent for the retrospective records review, data collection, and subsequent deidentified data analysis for individuals for whom Sources of Distress was completed as part of their clinical care (IRB_00170868).

### Early Survey Development

Funding for the development of Sources of Distress was provided by the Autism Council of Utah based in Murray, Utah, United States [41]. The development team comprised a triple board physician (pediatrics, general psychiatry, and child and adolescent psychiatry), an educational psychologist, a medical student, and a business consultant grandparent of a child with autism and ID. In the initial development phase, Sources of Distress was built in Qualtrics (Qualtrics International Inc) using a branching logic format to approximate the history-taking component of a DD psychiatric evaluation. This evaluation queries psychiatric symptom clusters, physical complaints, and psychiatric medical history to support the development of a diagnostic impression for which treatment recommendations could be made.

Multiple expert opinion sources were reviewed to identify pertinent screening categories and corresponding items to include in Sources of Distress. The expert sources included published literature, the *DM-ID-2*, the Mini PAS-ADD Clinical Interview, and the screening interview for the Kiddie Schedule for Affective Disorders and Schizophrenia–Present and Lifetime (a semistructured psychiatric diagnostic interview for children and adolescents) [28,32,42]. As Sources of Distress is intended for use in the context of distress, the presence of at least 1 manifestation of a behavioral or emotional crisis must be endorsed to initiate survey questions.

### Initial Survey Evaluation

#### Focus Group Evaluation

In 2018 and early 2019, focus group participants were recruited from (1) a university-based outpatient program that provides medical and psychiatric care for individuals with DD across the lifespan and (2) the Autism Council of Utah (a community stakeholder organization for individuals and families affected by autism). Six focus groups were conducted that consisted collectively of parents (6/17, 35%), professional caregivers (6/17, 35%), and adults with both DD and the ability to provide verbal feedback (5/17, 29%). Participants completed Sources of Distress before attending the focus group and reported on specific items, missing items, item wording, and attribution of items to corresponding conditions. Interviews and discussions were transcribed and analyzed following the framework analysis of Ritchie and Spencer [43]. Inductive reasoning and the constant comparative method put forth by Strauss and Corbin [44] were used to compare statements by parents, professional caregivers, and individuals with disability within and across focus groups.

#### Expert Review Evaluation

Revisions were made to Sources of Distress based on focus group feedback. Experts reviewed the revised survey version, and additional changes were made. The experts included a pediatrician and 2 child psychiatrists, all with national recognition for their clinical and research work in DD.

### Clinical Implementation

#### Overview

Sources of Distress was implemented in various clinical settings to augment the clinical history-taking process—outpatient (primary care, neurology, developmental pediatrics, and psychiatry), emergency department, psychiatric inpatient, and residential care. Caregivers were given a link to the survey when their health care provider identified the need for expert support in managing severe agitation and aggression. All caregivers (231/231, 100%) completed the survey outside of the clinical setting. An informal or formal consult followed survey completion for a subset of individuals. In August 2020, the survey was transitioned from the Qualtrics platform to the REDCap (Research Electronic Data Capture; Vanderbilt University) platform to automate the Sources of Distress report generation using the custom template engine [45]. This external REDCap module was developed and has been maintained by the Integrated Research Informatics Services of British Columbia Children’s Hospital Research Institute [46].

#### Survey Data Collection

Sources of Distress responses were collected from its first use in a clinical setting from February 2019 through June 2022. The following information was obtained: respondent type, individual characteristics, caregiver-reported diagnoses, current medications, distress manifestations, psychiatric symptoms, and medical symptoms, conditions, or concerns. When multiple caregivers reported on the same individual, responses were used from the caregiver closest to where the individual lived (eg, parent for a child living at home and professional caregiver for an individual living in a residential setting). Psychotropic medications were organized within the following mutually exclusive categories: antipsychotics, antidepressants, non-antidepressant anxiolytics, anticonvulsants, lithium, alpha-2 agonists, stimulants, and atomoxetine.

#### Consults

A medical decision-making support consultation took place after survey completion as either an informal or a formal consult for a subset of individuals. This consult was conducted by a clinical team led by the triple board physician member of the survey’s development team. The consult team used *DM-ID-2* criteria as the basis for establishing psychiatric diagnoses. At a minimum (as an informal consult), the consult involved a discussion between a DD clinical expert and the referring provider. This discussion resulted in a collective determination of working diagnoses and treatment plan. A formal consult included the additional components of medical records review, caregiver interview, and direct participant evaluation. Psychiatric diagnoses that were not reported in the survey but discussed by the provider or documented in the medical record were included among preexisting diagnoses.

Working diagnoses were abstracted from formal and informal consult documentation and served as the standard to define true case status.

#### Mood Disorder Classification

The presence of a mood disorder among preexisting and working diagnoses was classified into mutually exclusive categories such that there was no overlap among individuals across mood disorder categories to allow for direct comparisons across preexisting diagnoses, survey screening status results, and working diagnoses. The following mood disorder classification hierarchy was used from highest to lowest: (1) episodic expansive mood, hypomania, mania, and bipolar disorder, hereafter collectively referred to as *bipolar disorder*, (2) disruptive mood dysregulation disorder (DMDD) and unspecified mood disorder, and (3) unipolar depression. If an individual had a diagnosis of bipolar disorder, regardless of what other mood disorder diagnosis was reported or identified, their mood disorder classification would be bipolar disorder. An individual was only classified with unipolar depression if (1) they had a depression diagnosis and (2) they had no other mood disorder diagnosis.

#### Statistical Analyses and Evaluation of Validity

Descriptive statistics and chi-square tests were conducted in SPSS (version 28.0; IBM Corp) with an α of .05 selected to assess statistical significance. Differences between surveys with an accompanying consult and those without were measured. Positive predictive value (PPV), negative predictive value (NPV), and accuracy rates were calculated for (1) preexisting diagnoses and (2) survey screening results with working diagnoses used as the determinant of true case status. We calculated 95% CIs for the binomial distribution of accuracy rates.

## Results

### Early Survey Development

Table 1 lists the modules and corresponding items initially selected as the categories, characteristics, and symptoms to be queried by Sources of Distress. The initial version of the survey included scoring algorithms to determine positive screen status for the following conditions: anxiety, unipolar depression, bipolar disorder, psychosis, and attention-deficit/hyperactivity disorder (ADHD).

**Table 1:** Description of Sources of Distress and additions in response to focus group feedback.

Module	Original items	Added in response to feedback
Introduction and demographics	Respondent’s relationship to the individual who is affected Distress symptoms Language ability Age Known diagnoses Current medications	For professional caregivers: how long have you known the affected individual? Added “increased fixation on certain things” and “changes in behavior such as increased isolation, social withdrawal” to distress symptoms Is there a difference in language ability at the physician’s office? If so, is there something the provider can do to improve the individual’s ability to speak for themselves?
Behavior patterns and triggers	Circumstances of disruptive behavior (recognized triggers, patterns, motivation and reinforcement, and location)	Query perceived function to behavior surrounding distress
Sleep	Time of sleep onset and awakening Middle-of-the-night interruptions Naps Activities interfering with sleep onset or returning to sleep Intermittent periods of decreased need for sleep	Food seeking as an activity interfering with sleep Sleep apnea diagnosis and symptoms Discomfort precipitating sleep disturbance
Anxiety	Leading to significant outbursts or discomfort: transitioning activities, getting stuck on certain topics or things, and minor changes in daily activities	Panic and nightmares Sensory sensitivity that leads to discomfort Repeated checking or rituals, which interferes with daily activities
Depression	Less energy than usual, increased crying spells, sadness, irritability, isolative, loss of interest in activities typically enjoyed, and excess sleep Injures self on purpose; if yes: location of injury and whether self-injury is causing discomfort?	Whether self-injury is concerning to parent or caregiver Whether self-injury could be perpetuated by attention seeking or avoidance
Mania	Establish baseline energy Query discrete periods out of the blue lasting ≥2 days of increased energy compared to baseline, laughing or vocalizing for no clear reason, particularly happy or giddy, risk taking, sexually acting out, increased impulsivity, and decreased need for sleep	No changes made
Psychosis	Appearing to be responding to internal auditory or visual stimuli Yelling angrily in a room where no one else is present as if yelling at someone who is not there	No changes made
ADHD^a^	Difficulty following through on instructions, avoiding task demands, easily distractible, fidgety or restless, high activity when expected to remain in 1 place, constantly moving, blurting into other people’s conversations, and demanding attention or desired items	Excessive talking
General medical problems	Query history of headaches, seizures, injuries that can be causing discomfort, thyroid abnormalities, and tooth pain Could any of these issues be contributing to distress?	Are there unusual ways of responding to physical discomfort? Added joint pain; ear, nose, or throat pain; and seasonal allergies
Trauma^b^	N/A^c^	History of trauma Related to trauma: avoidance, flashbacks, and nightmares Hypervigilance
Gastrointestinal concerns^b^	N/A	Bowel movement frequency Query history of constipation, stool accidents, frequent stomachaches, food allergies, and acid reflux. Could any of these issues contribute to distress? Subsequent additions: changes in appetite, nausea, and variable bowel movements
Menstrual concerns^b^ (for female patients only)	N/A	Query presence of mood changes during menses, endometriosis, polycystic ovary syndrome, significant menstrual pain, excess bleeding during or between cycles, and anxiety surrounding periods. Could any of these conditions be leading to distress? Birth control: oral contraceptives, hormonal IUD^d^, nonhormonal IUD, and Depo-Provera (a contraceptive injection).
Dental concerns^b^	N/A	When was the last dental visit? Query presence of changes in eating patterns: texture preference, sensitivity to hot or cold food or drink preference for eating on 1 side of the mouth, and reduced oral intake Grinding teeth

^a^ADHD: attention-deficit/hyperactivity disorder.

^b^Module added in response to focus group feedback.

^c^N/A: not applicable.

^d^IUD: intrauterine device.

### Initial Survey Evaluation

#### Focus Group Feedback

During the focus groups, 3 main themes emerged in this analysis.

- Theme A: respondents gave overall positive feedback regarding existing content and specific feedback regarding areas where there was room to expand content. Table 1 describes the modules and items added in response to this feedback. Notably, a posttraumatic stress disorder (PTSD) module was added along with a PTSD scoring algorithm to determine positive screen status.
- Theme B: most of the respondents (15/17, 88%) agreed that the symptoms queried matched their understanding of the psychiatric and medical conditions to which they are attributed.
- Theme C: all participant groups reported positive acceptability of the branching logic format and time required to complete the measure.

#### Expert Review

Overall, the expert review supported the Sources of Distress categories and respective items attributed to each condition. One expert recommended adding items that query gender and replacing sex as the basis for pronoun selection within the tool and its report. This expert also suggested that the report include screening results for each psychiatric condition. The former recommendations were implemented when Sources of Distress was transitioned to the REDCap platform. The latter recommendation was deferred until after screening algorithms are validated in a clinical setting.

### Clinical Implementation

#### Sample Characteristics

Surveys (N=264) were completed by parents or guardians (n=200, 75.8%), professional caregivers (n=43, 16.3%), and other caregivers (n=21, 8%) of 231 individuals (n=161, 69.7% men and boys; n=69, 29.9% women and girls; and n=1, 0.4% other; mean age 17.7, SD 10.3; range 2-65 years). Informal (n=62, 41.6%) and formal (n=87, 58.4%) consults were performed for 149 individuals collectively. Table 2 presents sample characteristics, the manifestations of distress, and a comparison between individuals with a consult and those without.

**Table 2:** Sample characteristics and distress manifestations.

Characteristics		With consult^a^ (n=149), n (%)	Without consult (n=82), n (%)	Total (N=231), n (%)	Chi-square (*df*)		*P* value	
**Gender^b^**						2.3 (2)		.34
	Man or boy	102 (68.5)	59 (72)	161 (69.7)				
	Woman or girl	47 (31.5)	22 (25.5)	69 (29.9)				
	Other^b^	0 (0)	1 (1.2)	1 (0.4)				
**Caregiver^c^**						7.8 (2)		.02
	Parent or guardian	108 (72.5)	71 (86.6)	179 (77.5)				
	Professional caregiver	32 (21.5)	6 (7.3)	38 (16.5)				
	Other	9 (6)	5 (6.1)	14 (6.1)				
**Age range (y)**						7.4 (2)		.03
	<13	46 (30.9)	38 (46.3)	84 (36.4)				
	13-22	57 (38.3)	30 (36.6)	87 (37.7)				
	>22	46 (30.9)	14 (17.1)	60 (26)				
**Language ability**						0.0 (2)		.99
	Full verbal ability	76 (51)	42 (51.2)	118 (51.1)				
	Limited use of words	46 (30.9)	25 (30.5)	71 (30.7)				
	Nonverbal	27 (18.1)	15 (18.3)	42 (18.2)				
**Manifestation of distress**								
	Agitation	130 (87.2)	70 (85.4)	200 (86.6)	0.2 (1)		.69	
	Aggression	97 (65.1)	50 (61)	147 (63.6)	0.4 (1)		.53	
	Change in sleep	86 (57.7)	38 (46.3)	124 (53.7)	2.8 (1)		.10	
	Moodiness	122 (81.9)	65 (79.3)	187 (81)	0.2 (1)		.63	
	Increased fixation	115 (77.2)	57 (69.5)	172 (74.5)	1.6 (1)		.20	
	Change in eating patterns	52 (34.9)	19 (23.2)	71 (30.7)	3.4 (1)		.07	
	Change in personality	99 (66.4)	59 (72.0)	158 (68.4)	0.7 (1)		.39	
	Change in behavior	96 (64.4)	45 (54.9)	141 (61)	2.0 (1)		.15	
	Self-injurious behavior	73 (49)	39 (47.6)	112 (48.5)	0.0 (1)		.84	
**Type of disability**								
	Autism without ID^d^	52 (34.9)	47 (57.3)	99 (42.9)	10.9 (1)		<.001	
	ID without autism	15 (10.1)	7 (8.5)	22 (9.5)	0.1 (1)		.71	
	ID and autism	74 (49.7)	18 (22.0)	92 (39.8)	17.0 (1)		<.001	
	Genetic syndrome^e^	17 (11)	17 (20.7)	34 (14.7)	3.7 (1)		.06	

^a^Includes informal and formal consults.

^b^One participant reported *other* as gender: no participants reported *non-binary* as gender.

^c^When multiple caregivers completed Sources of Distress, the report from the caregiver with whom the participant spends the most time was used in this table.

^d^ID: intellectual disability.

^e^Genetic syndrome includes some individuals who also populate the autism or ID categories.

#### Preexisting Psychiatric Diagnoses

The presence of at least 1 preexisting psychiatric diagnosis was reported in 65.4% (151/231) of the individuals. Individuals who received a consult compared to those without a consult were more likely to have a caregiver-reported history of psychotic disorder (14/149, 9.4% vs 1/82, 1%; *P*=.02; Table 3).

**Table 3:** Medical conditions, preexisting psychiatric diagnoses, and psychiatric screening results.

Characteristics					With consult^a^ (n=149), n (%)			Without consult (n=82), n (%)			Total (N=231), n (%)			Chi-square (*df*)			*P* value
**Medical conditions^b^**																	
	Gastrointestinal concerns			82 (55)			37 (45.1)			119 (51.5)			2.0 (1)			.15	
	Dental concerns			32 (21.5)			25 (30.5)			57 (24.7)			2.3 (1)			.13	
	Menstrual concerns^c^			16 (53.3)			5 (27.8)			21 (43.8)			3.0 (1)			.13	
	**General**																
		Headache	26 (17.4)			8 (9.9)			34 (14.7)			2.5 (1)			.11		
		Ear, nose, and throat concerns	17 (11.4)			8 (9.9)			25 (10.8)			0.2 (1)			.70		
		Seasonal allergies	34 (22.8)			13 (15.9)			47 (20.3)			1.6 (1)			.21		
		Injury pain	14 (9.4)			9 (11)			23 (10)			0.2 (1)			.70		
		Thyroid abnormalities	5 (3.4)			6 (7.3)			11 (4.8)			1.8 (1)			.18		
		Joint pain	9 (6)			3 (3.7)			12 (5.2)			0.6 (1)			.44		
		Seizures	29 (19.5)			16 (19.5)			45 (19.5)			0.0 (1)			.99		
Seizure History					40 (26.8)			18 (22)			58 (25.1)			0.7 (1)			.41
Sleep disturbance					124 (83.2)			68 (82.9)			192 (83.1)			0.0 (1)			.95
**Preexisting psychiatric diagnoses**																	
	Any psychiatric condition			103 (69.1)			48 (58.5)			151 (65.4)			2.6 (1)			.11	
	Depression^d^			20 (13.4)			14 (17.1)			34 (14.7)			0.6 (1)			.46	
	Bipolar disorder^d^			21 (14.1)			9 (11)			30 (13)			0.5 (1)			.50	
	Unspecified mood disorder or DMDD^d,e^			20 (13.4)			7 (8.5)			27 (11.7)			1.2 (1)			.27	
	Anxiety^f^			62 (41.6)			31 (37.8)			93 (40.3)			0.3 (1)			.57	
	PTSD^g^			11 (7.4)			4 (4.9)			15 (6.5)			0.6 (1)			.46	
	Psychotic disorder			14 (9.4)			1 (1.2)			15 (6.5)			5.8 (1)			.02	
	ADHD^h^			50 (33.6)			26 (31.7)			76 (32.9)			0.1 (1)			.78	
**Psychiatric screening status**																	
	Any psychiatric condition			146 (98)			80 (97.6)			226 (97.8)			0.1 (1)			.83	
	Unipolar depression^d^			61 (40.9)			30 (36.6)			91 (39.4)			0.4 (1)			.52	
	Episodic expansive mood and bipolar disorder^d^			60 (40.3)			28 (34.1)			88 (38.1)			0.8 (1)			.36	
	Anxiety			130 (87.2)			71 (86.6)			201 (87)			0.0 (1)			.89	
	PTSD			37 (24.8)			15 (18.3)			52 (22.5)			1.3 (1)			.26	
	Psychosis			52 (34.9)			15 (18.3)			67 (29)			7.1 (1)			.008	
	ADHD			102 (68.5)			56 (68.3)			158 (68.4)			0.0 (1)			.98	

^a^Includes informal and formal consults.

^b^Medical conditions perceived by the caregiver as contributing to the current presentation of distress.

^c^Analysis for menstrual concerns restricted to female patients aged >12.

^d^Unipolar depression, unspecified mood disorder and disruptive mood dysregulation disorder, and episodic expansive mood and bipolar disorder are mutually exclusive categories.

^e^DMDD: disruptive mood dysregulation disorder.

^f^Preexisting diagnosis of obsessive-compulsive disorder is included within the anxiety disorder category.

^g^PTSD: posttraumatic stress disorder.

^h^ADHD: attention-deficit/hyperactivity disorder.

#### Caregiver-Reported Medical Conditions

Table 3 describes medical conditions reported by caregivers. Caregivers of 73.2% (169/231) of the individuals identified at least 1 physical concern that they perceived as contributing to distress. The most common conditions were gastrointestinal concerns (119/231, 51.5%), menstrual concerns (21/48, 44% of female patients aged >12 y), seasonal allergies (47/231, 20.3%), and seizures (45/231, 19.5%).

#### Psychiatric Screening Results

Table 3 lists the frequency of positive psychiatric screening results. All but 2% (5/231) of the individuals screened positive for a psychiatric condition, with a mean of 2.8 (SD 1.1; range 0-5) conditions per individual. Of those who were classified as having bipolar disorder, 89% (78/88) screened positive for a recent depressive episode. Positive screen status for psychiatric conditions were similar between those with a consult and those without, except in the case of psychosis (52/149, 34.9% vs 15/82, 18%; *P*=.008).

#### Psychotropic Medication Use

Table 4 reports on the frequency of medication use. Most of the individuals (194/231, 84%) were taking psychotropic medication, and the majority were receiving antipsychotics (142/231, 61.5%) and antidepressants (129/231, 55.8%).

**Table 4:** Medication use reported in Sources of Distress.

Medication		With consult (n=149), n (%)	Without consult (n=82), n (%)	Total (N=231), n (%)	Chi-square (*df*)	*P* value
Any medication		146 (98)	68 (82.9)	214 (92.6)	17.6 (1)	<.001
**Any psychotropic medication**		136 (91.3)	58 (70.7)	194 (84)	16.6 (1)	<.001
	Antipsychotic	102 (68.5)	40 (48.8)	142 (61.5)	8.7 (1)	.003
	Antidepressant^a^	87 (58.4)	42 (51.2)	129 (55.8)	1.1 (1)	.29
	Anxiolytic^b^	66 (44.3)	23 (28.0)	89 (38.5)	5.9 (1)	.02
	Anticonvulsant^c^	45 (30.2)	12 (14.6)	57 (24.7)	6.9 (1)	.009
	Lithium	10 (6.7)	7 (8.5)	17 (7.4)	0.3 (1)	.61
	Alpha-2 agonist	72 (48.3)	27 (32.9)	100 (43.3)	5.1 (1)	.02
	Stimulant and atomoxetine	30 (20.1)	14 (17.1)	44 (19)	0.3 (1)	.57

^a^Selective serotonin reuptake inhibitors, duloxetine, tricyclics, mirtazapine, and trazodone were included exclusively within the antidepressant category.

^b^Benzodiazapines, buspirone, hydroxyzine, beta-blockers, and prazosin were included exclusively within the anxiolytic category.

^c^Anticonvulsant medication use in the absence of a reported seizure history.

#### Working Psychiatric Diagnoses

Of the 149 individuals who received a consult, 148 (99.3%) were diagnosed with at least 1 psychiatric condition with a mean of 2.7 (SD 1.0; range 0-5) diagnoses per individual. The conditions identified were anxiety (129/149, 86.6%), ADHD (84/149, 56.4%), bipolar disorder (67/149, 45%), unipolar depression (33/149, 22.1%), PTSD (35/149, 23.5%), and psychosis (31/149, 20.8%). Furthermore, 25 (16.8%) of the 149 individuals were diagnosed with either unspecified mood disorder or DMDD. Nearly all individuals identified with psychosis (29/31, 94%) had a co-occurring mood disorder diagnosis: bipolar disorder (22/31, 71%), unipolar depression (5/31, 16%), and unspecified mood disorder or DMDD (2/31, 6%).

#### Evaluation of Validity

Sources of Distress accuracy rates ranged from 76% (95% CI 69%-82%) for ADHD to 91% (95% CI 85%-95%) for PTSD and exceeded those of preexisting diagnoses, except in the case of psychosis, for which the accuracy rates were equivocal (82%, 95% CI 75%-87%; Table 5). The survey demonstrated higher NPVs (81%-98%) than PPVs (51%-78%) for all conditions, with the exceptions of anxiety (53% and 92%, respectively) and episodic expansive mood bipolar disorder (85% and 90%, respectively). Low PPVs were notable for depression (51%) and psychosis (54%).

**Table 5:** Association between consult diagnoses after completing Sources of Distress with preexisting psychiatric diagnoses and Sources of Distress screening status (n=149).

Working diagnosis			Preexisting psychiatric diagnosis^a^															Sources of Distress screening status												
			Case negative, n		Case positive, n		PPV^b^ (%)			NPV^c^ (%)			Accuracy rate^d^, % (95% CI)			Screen negative, n				Screen positive, n			PPV (%)^b^			NPV (%)^c^			Accuracy rate^d^, % (95% CI)	
**Unipolar depression^e,f^**								50			82			78 (71-84)										51			98			79 (71-85)
	Case negative	106		10											86				30											
	Case positive	23		10											2				31											
**Episodic expansive mood and bipolar disorder^e,f^**								76			60			62 (55-70)										90			85			87 (81-92)
	Case negative	77		5											76				6											
	Case positive	51		16											13				54											
**DMDD^g^ and unspecified mood disorder^e^**								45			88			82 (75-88)										N/A^h^			N/A			N/A
	Case negative	113		11											N/A				N/A											
	Case positive	16		9											N/A				N/A											
**Anxiety disorder^i^**								97			20			52 (44-60)										92			53			87 (81-92)
	Case negative	18		2											10				10											
	Case positive	69		60											9				120											
**Posttraumatic stress disorder**								100			83			84 (77-89)										78			95			91 (85-95)
	Case negative	114		0											106				8											
	Case positive	24		11											6				29											
**Psychotic disorder^j^**								64			84			82 (75-87)										54			97			82 (75-87)
	Case negative	113		5											94				24											
	Case positive	22		9											3				28											
**Attention-deficit/hyperactivity disorder**								84			58			66 (59-74)										74			81			76 (69-82)
	Case negative	57		8											38				27											
	Case positive	42		42											9				75											

^a^Preexisting diagnoses included caregiver-reported diagnoses in Sources of Distress and diagnoses in the medical record before survey completion.

^b^PPV: positive predictive value = 
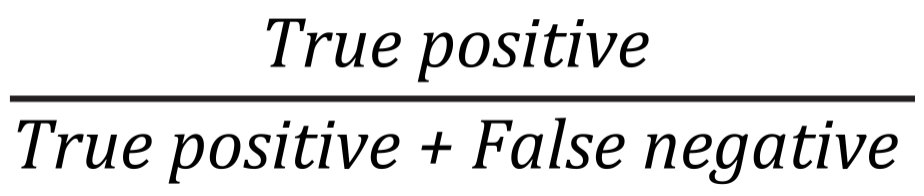

^c^NPV: negative predictive value = 
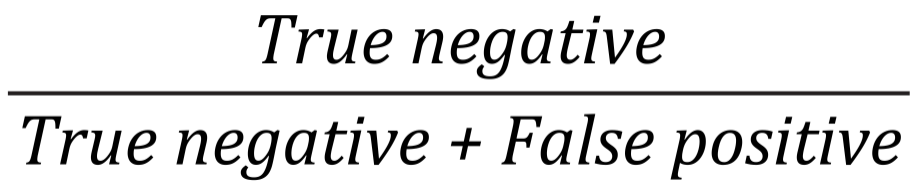

^d^Accuracy rate = 
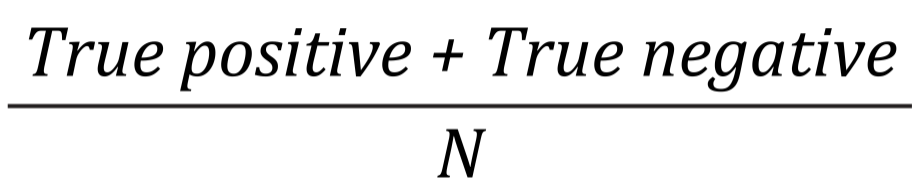

^e^Depression, episodic expansive mood and bipolar disorder, and disruptive mood dysregulation disorder and unspecified mood disorder are mutually exclusive categories. There is no Sources of Distress screening algorithm for disruptive mood dysregulation disorder or unspecified mood disorder.

^f^Preexisting and working diagnoses included schizoaffective disorder when hypomanic, manic, or mixed episode was specified.

^g^DMDD: disruptive mood dysregulation disorder.

^h^N/A: not applicable.

^i^Preexisting and working diagnoses of anxiety disorder and obsessive-compulsive disorder are combined to coincide with anxiety disorder screening status.

^j^Preexisting and working diagnoses were schizophrenia, schizoaffective disorder, unspecified psychotic disorder, and psychotic features associated with a mood disorder.

#### Exploration of Mood Disorder Categories

**Figure 1:**
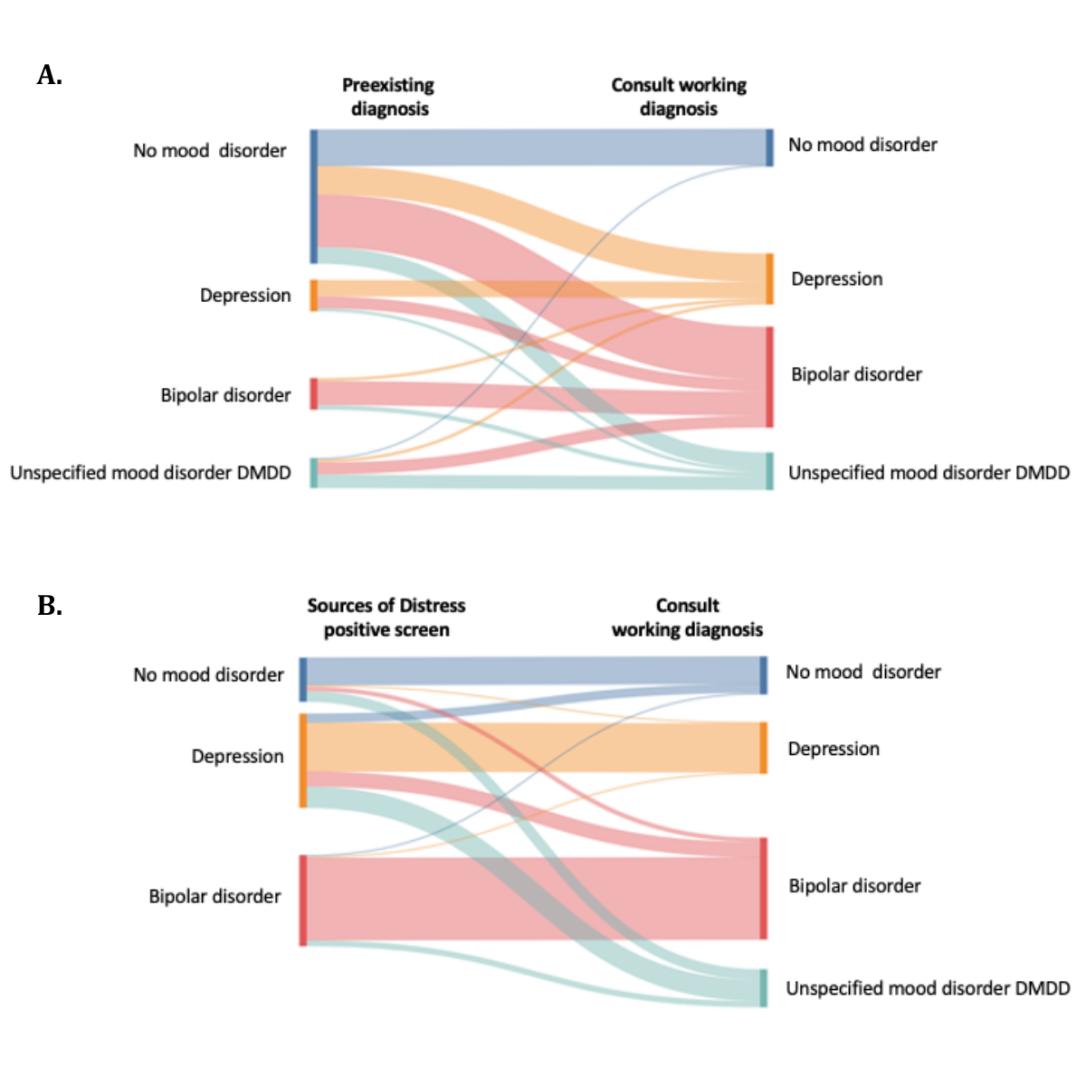
Comparison of mood disorder categorization between working diagnosis established during consultation and (A) preexisting diagnosis and (B) Sources of Distress positive screen. DMDD: disruptive mood dysregulation disorder.

Figure 1 demonstrates the distribution of mood disorder diagnoses among individuals based on (1) preexisting mood disorder diagnosis and (2) Sources of Distress mood disorder screening status. The majority of the individuals (18/25, 72%) who received a working diagnosis of unspecified mood disorder and DMDD screened positive for either unipolar depression or bipolar disorder.

## Discussion

### Principal Findings

The focus group feedback indicates that Sources of Distress provides an acceptable means for caregivers to share their knowledge and insights about individuals with DD who present in crisis. As a screening tool, this survey demonstrates good accuracy, although additional work is needed to differentiate among mood disorders. The purpose of this survey is to screen individuals with DD for mental health and common medical concerns in health care settings when they present in crisis. By querying what symptom clusters and physical conditions coincide with their patient’s crisis, providers can direct their evaluation toward specific psychiatric and medical conditions that have established treatment protocols in the general population. This approach aims to reduce diagnostic overshadowing and improve medical decision-making surrounding the management of agitation and aggression in individuals with DD. Focus group participants validated the survey content and provided recommendations that prompted the inclusion of additional modules and items. Despite its length (ie, 15-20 min), participants reported positive acceptability of the survey’s format and duration. This feedback may reflect the convenience of completing a web-based survey at home versus in the medical setting and highlights caregivers’ motivation toward understanding potential factors contributing to the person’s distress. After incorporating caregiver recommendations, Sources of Distress content was also reviewed and supported by clinical and research experts.

Caregivers of most of the individuals (200/231, 86.6%) identified agitation as a presenting concern. The Food and Drug Administration has approved short-term antipsychotic medication for treating irritability in individuals with autism [47]; 61.5% (142/231) of the individuals were taking antipsychotics at the time of presenting in crisis. This frequency exceeds previously reported estimates of antipsychotic use in the population with DD (ie, 10%-48%) and reflects the high acuity and potentially treatment-resistant nature of individuals for whom the survey was completed [48,49]. This study group’s acuity is further supported by the high frequency in which severe mental health conditions were diagnosed in those receiving a consultation (eg, bipolar disorder and psychosis).

Anxiety was the most common condition to screen positive (201/231, 87%) and be established as a working diagnosis (129/149, 86.6%). These rates exceeded measured anxiety prevalence rates in the population with DD (ie, 20%-77%), indicating a higher propensity toward experiencing anxiety among those presenting in crisis [12,15,16]. As a precipitant of distress, prior studies have identified aggression, disruptive behavior, sleep disturbance, and self-injurious behavior as symptoms of anxiety among individuals with DD [4,8,50]. To reduce overclassification among individuals whose autism core features overlap with some anxiety symptoms [51], the Sources of Distress anxiety scoring algorithm was set at a higher threshold than the generalized anxiety disorder criteria described in *DM-ID-2*. The survey’s low NPV (53%) and high PPV (92%) for anxiety likely reflect this adaption.

Sources of Distress captured well the presence of a mood disturbance; however, the type of mood disorder was not. Study results report a diagnosis frequency of 16.8% (25/149) for unspecified mood disorder and DMDD and indicate the need to add items and a screening algorithm for this condition. The low PPV (51%) for depression primarily resulted from individuals screening positive for depression who were subsequently diagnosed with unspecified mood disorder and DMDD. The *DM-ID-2*, survey data, and records review will inform new items and algorithm development as well as revisions for the depression screening algorithm. In the interim, the Sources of Distress report will replace the “depression” category label with “depression and unspecified mood disorder” to broaden the range of conditions which it currently captures.

Caregivers of the majority of the individuals (169/231, 73.2%) identified at least 1 physical concern that they perceived as contributing to distress. As agitation may be one of the few visible indicators of pain in an individual with limited expressive language ability and DD, sources of pain should be considered when unexplained agitation is present [3,9,52]. Limited access to medical care by the population with DD further reduces the likelihood that pain and other underlying physical causes of agitation are recognized [53]. Through Sources of Distress, caregivers demonstrated their ability to provide meaningful insight into the potential presence of physical discomfort. This attention was directed most frequently to gastrointestinal, menstrual, dental, and seizure concerns.

### Limitations

The generalizability of study results is limited to the geographic, racial, and ethnic diversity of Utah. While survey access requires internet or smartphone access, it has been completed by parents without this access through the assistance of state-sponsored support coordinators and medical assistants. Sources of Distress has a Spanish translation available (*Causas de Aflicción*); however, these data were not included because its content has not yet been validated by Spanish-speaking caregivers and individuals who are affected. The expert leading the consult team was a member of the survey’s development team, which introduces the inherent bias of evaluating for the presence of mental health conditions through the lens of *DM-ID-2* criteria on which survey components were also based. While the *DM-ID-2* is well recognized and accepted in the ID provider community, few autism specialty providers are familiar with its use.

### Future Directions

Edits and additions to mood disorder items and scoring algorithms are being made to improve differentiation across mood disorders. Branching logic that incorporates the individual’s language ability has recently been added to the psychosis module to improve question clarity and scoring algorithm accuracy. The most updated version of the Sources of Distress can be accessed through the Utah Department of Health and Human Services Autism Systems Development Program webpage [39]. Reevaluation of the survey’s PPVs, NPVs, and accuracy will follow the completion of these changes. Additional studies of this survey are needed to measure its acceptability and validity in clinical settings outside of Utah and by other DD specialty providers. REDCap has also demonstrated capacity to integrate digital mental health screening results into electronic medical records, significantly improving provider adoption of the screening tools [54]. The integration of Sources of Distress into electronic medical records could further enhance its impact on provider efficiency. This survey has already been used during medical evaluations to facilitate the consideration of potential discordance between medications prescribed and conditions present [55]. Prospective studies are merited to determine the survey’s impact on treatment approaches, hospital and emergency department use, and outcomes for individuals with DD who experience crisis.

### Conclusions

Individuals with DD presenting in crisis experience high rates of psychiatric disorders and medical concerns that may contribute to, or manifest as, distress. Sources of Distress is a valuable screening tool for psychiatric and medical conditions that commonly accompany treatment-resistant agitation in individuals with DD. When systematically queried, caregivers’ knowledge provides essential information to minimize diagnostic overshadowing and support an evaluation focused on the individual rather than their disability when persistent agitation is assessed in the population with DD.

## Appendix

Multimedia Appendix 1 A sample of the Sources of Distress report.

## Abbreviations

ADHD: attention-deficit/hyperactivity disorder
DD: developmental disabilities
DMDD: disruptive mood dysregulation disorder
DM-ID-2: Diagnostic Manual–Intellectual Disability–Second Edition
ID: intellectual disability
NPV: negative predictive value
PAS-ADD: Psychiatric Assessment Schedule for Adults with Developmental Disabilities
PPV: positive predictive value
PTSD: posttraumatic stress disorder
REDCap: Research Electronic Data Capture
