# Supplementary material for: Developing and Implementing a Web-Based Branching Logic Survey to Support Psychiatric Crisis Evaluations of Individuals With Developmental Disabilities: Qualitative Study and Evaluation of Validity
Source: JMIR Ment Health. 2024 Mar 29;11:e50907. doi: 10.2196/50907 (PMC11015367; doi:10.2196/50907)
Supplement: Multimedia Appendix 1 [file mental_v11i1e50907_app1.pdf]

Sample Sources of Distress Report

Distress can manifest in several ways in people with developmental disabilities.

For Johnny, distress may be causing the following symptoms you described: agitation (Ex: physical/verbal aggression, property destruction, etc.), aggression, changes in personality, changes in eating, moodiness, increased fixation on certain topics or things.

Common sources of distress include anxiety, depression, mania, inattention/hyperactivity/impulsivity, sleep disturbance, physical discomfort, gastrointestinal problems, dental issues, and uncontrolled seizures.

Below are the items that you, as Johnny's parent, endorsed for Johnny that correspond to symptoms of conditions commonly experienced by people with developmental disabilities. Diagnoses are established by a trained provider. The information you shared below, along with further clarification from you and Johnny may help your provider establish a working diagnosis. Your provider will also observe Johnny and may need to do a physical exam based the concerns you have raised to aid in making this diagnosis. The purpose of Sources of Distress is to facilitate accurate recognition of what contributes to Johnny's distress so that these conditions are treated as effectively and safely as possible.

Johnny is 8 years old and has the following language ability: full verbal ability to express self.

Current diagnoses: Autism, aggression

Current medications are: Risperidone 0.25 mg BID

Johnny's experience of distress is disruptive to those around him.
You have noticed the following triggers for Johnny: Poor sleep, if Johnny feels socially uncomfortable, if told "no". Behaviors may be motivated by attention seeking, avoiding a task or demand, trying to obtain a desired item or get to do a desired activity. Distress occurs in the school/work, community, home.

Sleep Disturbance
Johnny sleeps from 11 pm to 8 am, and sleeps 7-9 hours a night, and does not take naps.
Johnny does not wake up in the middle of the night.
Johnny has no difficulties with sleep.

Anxiety
Johnny experiences outbursts related to transitioning between activities.
Johnny gets stuck on certain topics or things, which causes significant disruption or discomfort.
Johnny is unable to tolerate minor changes in daily activities, which causes significant disruption or discomfort.
Johnny experiences sudden onset of anxiety or panic.
Johnny experiences high sensory sensitivity/sensory seeking.

Post-Traumatic Stress Disorder
Johnny has no known history of significant trauma or abuse.

Depression
Johnny experiences the following symptoms of depression: much less energy than usual, seeking isolation more than usual, losing interest in activities that previously were enjoyed.

Mania
Compared to peers with typical development, at baseline Johnny's level of energy is average.
Johnny has no current or previous symptoms of mania.

Inattention/hyperactivity/impulsivity (ADHD)
Johnny experiences the following symptoms of ADHD: easily distracted, blurting out answers before being called upon or interrupting others in conversation, excessively talking.

Psychosis
Johnny has no apparent symptoms of psychosis.

Medical Issues
Johnny has no history of headaches, seizures, injuries that can be causing discomfort, thyroid abnormalities, joint pain, ear/noise/throat pain, or seasonal allergies.
Johnny has a full bowel movement every 1-2 days.
Johnny has a history of the following abnormalities related to bowel movements: constipation. You feel it is possible that this/these condition(s) could be leading to distress.
Johnny has a history of the following: acid reflux, appetite changes. You feel it is possible that this/these condition(s) could be leading to distress.

Dental issues
Johnny's last dental visit was less than 6 months ago.
Johnny has not experienced any changes in eating patterns.
Johnny does not grind his teeth.
You have no concerns about Johnny's dental health.

Additional information
Johnny is gaining a lot of weight and eating more than normal.

Reference:
The Sources of Distress Survey Tool is copyrighted but can be utilized at the Utah Department of Health Website. https://familyhealth.utah.gov/cshcn/asd/#

Sample Sources of Distress Report is a multimedia appendix to JMIR Mental Health Article: Bilder DA, Mthembu M, Worsham W, Aguayo P, Knight JR, Deng SW, Singh TP, Davis J Developing and Implementing a Web-Based Branching Logic Survey to Support Psychiatric Crisis Evaluations of Individuals With Developmental Disabilities: Qualitative Study and Evaluation of Validity JMIR Ment Health 2024;11:e50907 URL: https://mental.jmir.org/2024/1/e50907/ doi: 10.2196/50907
